# Pericapsular Nerve Group Block Versus Fascia Iliaca Compartment Block for Hip Arthroplasty: An Updated Meta‐Analysis of Randomized Controlled Trials With Trial Sequential Analysis

**DOI:** 10.1155/anrp/5550599

**Published:** 2026-05-12

**Authors:** Luigi La Via, Fabio Raciti, Giovanni Guarneri, Ornella Rosa, Francesco Vasile, Francesco Perna, Marco Sapienza, Carmelo Calvagna, Gianluca Testa, Vito Pavone

**Affiliations:** ^1^ Department of General Surgery and Medical-Surgical Specialties (CHIRMED), University of Catania, Catania, Italy, unict.it; ^2^ Department of Anesthesia and Intensive Care, University Hospital Policlinico “G. Rodolico – San Marco”, Catania, Italy; ^3^ Department of General Surgery and Medical-Surgical Specialties (CHIRMED), School of Orthopedic Surgery, University of Catania, Catania, Italy, unict.it; ^4^ Department of Anesthesia and Intensive Care, University “Magna Graecia”, Catanzaro, Italy, unicz.it; ^5^ Faculty of Medicine and Surgery, University of Catania, Catania, Italy, unict.it; ^6^ Department of Orthopedic Surgery, University Hospital Policlinico “G. Rodolico – San Marco”, Catania, Italy

**Keywords:** analgesia, enhanced recovery, mobilization, opioid sparing, PENG, rehabilitation

## Abstract

**Purpose:**

Regional anesthesia techniques are crucial for optimizing pain management after hip arthroplasty. The pericapsular nerve group (PENG) block represents a novel approach targeting specific neural structures for hip joint innervation.

**Objective:**

To evaluate the efficacy of PENG block compared to suprainguinal fascia iliaca compartment block (S‐FICB) in adult patients undergoing hip arthroplasty, focusing on postoperative opioid consumption, pain scores, and mobilization time.

**Methods:**

We conducted a systematic review following PRISMA guidelines, searching PubMed and Scopus databases through October 2025. Only randomized controlled trials comparing PENG block with S‐FICB were included. Risk of bias was assessed using the revised Cochrane tool. Meta‐analysis employed random effects modeling, with evidence strength evaluated using GRADE methodology.

**Results:**

Five randomized controlled trials (824 patients) were included. PENG block significantly reduced 24‐h opioid consumption compared to S‐FICB (MD −3.85 mg, 95% CI −6.61 to −1.09, *p* = 0.006, *I* ^2^ = 87%), improved pain scores (MD −0.74, 95% CI −1.28 to −0.19, *p* = 0.008, *I* ^2^ = 74%), and shortened time to mobilization (MD −6.94 h, 95% CI −8.96 to −4.93, *p* < 0.00001, *I* ^2^ = 0%). Trial sequential analysis demonstrated robust evidence for mobilization time, with qualified support for analgesic outcomes due to incomplete information size and heterogeneity. Evidence certainty was high for mobilization time and moderate for opioid consumption and pain scores.

**Conclusions:**

PENG block provides statistically significant reductions in opioid consumption and pain scores, with robust and clinically meaningful earlier mobilization compared to S‐FICB in patients undergoing hip arthroplasty. These findings support incorporating PENG block into multimodal enhanced recovery pathways, though heterogeneity in analgesic outcomes warrants cautious interpretation.

## 1. Introduction

Hip arthroplasty, including total hip arthroplasty (THA) and hemiarthroplasty, is one of the most common orthopedic procedures worldwide, and its incidence has increased because of increasing demographic aging and surgical indications [1, 2]. Although these procedures significantly increase the quality of life of patients with fracture, severe hip osteoarthritis, avascular necrosis (AVN) and other degenerative diseases, patients may experience significant postoperative pain [3]. The poor management of acute postoperative discomfort could also lead to chronic postsurgical pain, prolonged hospital length of stay, delayed recovery and related additional healthcare costs [4, 5]. Historically, postoperative pain following hip arthroplasty has been treated mainly with systemic opioids combined with neuraxial approaches, including epidural analgesia [6]. However, opioid‐associated side effects (respiratory depression, PONV, constipation, and opioid addiction) and prolonged motor blockade from neuraxial anesthesia potentially reduce early mobilization, a fundamental component of ERAS pathways [7, 8]. For these reasons, we observed increased interest in peripheral nerve block approaches that provide efficient analgesia without affecting motor function. Among these novel approaches, the pericapsular nerve group (PENG) block is one of the most used [9]. PENG block, initially reported by Girón‐Arango et al. in 2018, targets the articular branches of the femoral and obturator/accessory obturator nerves innervating the anterior hip capsule [10]. This innovative technique was developed for site‐specific analgesia for the hip joint, sparing motor function and increasing early mobilization and physiotherapy rehabilitation [11]. PENG block and suprainguinal fascia iliaca compartment block (S‐FICB) differ fundamentally in their anatomical targets and theoretical coverage. PENG block specifically targets the articular branches of the femoral nerve, obturator nerve, and accessory obturator nerve as they course between the psoas tendon and the pubic ramus, providing focused analgesia to the anterior hip capsule while theoretically preserving motor function to the quadriceps and hip adductors [10, 11]. In contrast, S‐FICB deposits local anesthetic in the fascia iliaca compartment above the iliacus muscle at a suprainguinal level, targeting the lateral femoral cutaneous nerve, femoral nerve, and potentially the obturator nerve through cranial spread toward the lumbar plexus [12, 13]. While S‐FICB provides broader anesthetic coverage of the anterior and lateral thigh, it may result in quadriceps weakness due to femoral nerve motor blockade, potentially delaying mobilization [14]. This mechanistic difference forms the rationale for comparing these techniques in the context of enhanced recovery pathways, where motor‐sparing analgesia facilitating early mobilization represents a key therapeutic goal. Although theoretically appealing, the evidence for the efficacy of PENG block is conflicting and consists of papers that have used different methods, comparators, and endpoints [15, 16]. Prior systematic reviews have sought to consolidate existing data regarding multiple regional techniques for hip surgery [17]; however, PENG blocks, especially in the setting of hip arthroplasty, and related outcomes, such as opioid consumption, pain scores, and early mobilization, are lacking. Recently, Prakash et al. [18] published a systematic review and meta‐analysis comparing the PENG block with FICB in hip arthroplasty, including 12 randomized controlled trials (RCTs) with 644 patients. Their analysis demonstrated moderate certainty evidence for improved analgesia within 30 min and reduced opioid consumption at 24 h with PENG block but found no difference in pain scores at 24 h between the two techniques. However, their analysis included both suprainguinal and infrainguinal FICB approaches pooled together; did not incorporate trial sequential analysis (TSA) to assess the robustness and conclusiveness of findings; and the search was conducted through April 2023, prior to the publication of several recent high‐quality RCTs. S‐FICB represents another regional anesthetic technique that has gained prominence in hip surgery analgesia. First described by Hebbard et al. in 2011, S‐FICB targets the lateral femoral cutaneous, femoral, and potentially obturator nerves by depositing local anesthetic above the iliacus muscle in the fascia iliaca compartment at a suprainguinal level [12]. This approach provides analgesia to the anterior, lateral, and medial aspects of the thigh while generally preserving quadriceps strength, thus potentially facilitating earlier mobilization compared to traditional approaches [17]. Multiple studies have demonstrated the efficacy of S‐FICB in reducing opioid consumption and improving pain scores following hip surgery, making it a compelling alternative to conventional analgesic methods [18, 19]. Given the emergence of new evidence and methodological gaps in prior syntheses, the present updated systematic review and meta‐analysis was conducted to (1) incorporate recently published RCTs comparing PENG block with S‐FICB specifically; (2) assess the robustness and sufficiency of accumulated evidence through TSA; and (3) evaluate analgesic efficacy focusing on the clinically relevant 24‐h timepoint for postoperative opioid consumption, pain scores, and time to mobilization.

## 2. Materials and Methods

### 2.1. Search Strategy and Selection Criteria

This systematic review and meta‐analysis were conducted in accordance with the Preferred Reporting Items for Systematic Reviews and Meta‐Analyses (PRISMA) guidelines, and a PRISMA Checklist is provided separately (Supporting Information 1) [20]. The protocol was regularly registered at PROSPERO University of York (ID number: CRD420251163331; date of registration: 7th October 2025). A web‐based systematic advanced literature search was carried out across two databases (PubMed and Scopus) from inception up to the 7^th^ October 2025, to retrieve the highest number of relevant studies. A further search, not originally planned, was performed on Cochrane Library on the 15^th^ November 2025, together with an update of the original search on PubMed and Scopus. No date restrictions were applied. Our full search strategy included a combination of MeSH terms and free‐text keywords. This systematic review updates prior evidence syntheses [15] by (1) restricting the comparator to S‐FICB exclusively, given anatomical and clinical differences from infrainguinal approaches [16, 17]; (2) incorporating TSA to evaluate whether accumulated evidence is sufficient and conclusive; (3) including RCTs published after April 2023, ensuring incorporation of the most recent high‐quality evidence; and (4) performing sensitivity analyses based on single‐shot versus continuous catheter techniques. The following Boolean string was used: (“Pericapsular Nerve Group Block” OR “PENG block”) AND (“Arthroplasty, Replacement, Hip”[MeSH] OR “Hip Arthroplasty” OR “Hip Replacement” OR “Hip Prosthesis”) AND (“Pain, Postoperative”[MeSH] OR “postoperative pain” OR “Analgesia”[MeSH] OR “Opioid Consumption” OR “opioid use” OR “Length of Stay”[MeSH] OR “hospital stay” OR “Time to Mobilization” OR “early ambulation” OR “early mobilization” OR “Rehabilitation”[MeSH] OR “rehabilitation”). Two further searches were performed manually and independently by two authors, who also explored the list of references of the findings of the systematic search. While our protocol specified searches of electronic databases (PubMed, Scopus, Cochrane Library—which includes the Cochrane Central Register of Controlled Trials incorporating trial registry data), we did not conduct separate explicit searches of clinical trial registries such as ClinicalTrials.gov or the WHO International Clinical Trials Registry Platform (ICTRP).

Duplicates were removed, and all titles and abstracts were independently screened by two experienced authors according to the prespecified PICOS criteria (Supporting Information 2). Disagreements were resolved by consensus. The data were entered into a password‐protected database in Excel.

We included only RCTs that enrolled adult patients who underwent hip arthroplasty (either hemiarthroplasty or THA) due to osteoarthritis, AVN, proximal femoral fractures, or hip dysplasia. We compared the use of PENG block combined with general or neuraxial anesthesia to the use of S‐FICB. As a primary outcome, we analyzed postoperative opioid consumption within the first 24 h. We also considered pain scores at 24 h and the time to mobilization as secondary outcomes. Language restrictions were applied: we included only studies published in English. We excluded case reports, case series, retrospective studies, prospective non‐RCT studies, experimental animal studies, book chapters, reviews, editorials, and letters to the editor. Studies involving pediatric populations and those assessing the use of PENG block performed in the postoperative period or in patients not undergoing hip arthroplasty were also excluded.

### 2.2. Quality Assessment, Publication Bias, and GRADE of Evidence

To ensure the methodological quality of the included studies, the revised Cochrane Risk of Bias tool (RoB 2.0) was used, in accordance with PRISMA guidelines [20]. Each study was assessed across five specific domains: bias arising from the randomization process (D1), bias due to deviations from intended interventions (D2), bias due to missing outcome data (D3), bias in measurement of the outcome (D4), and bias in selection of the reported result (D5). Two reviewers independently assessed the risk of bias, and disagreements were resolved through discussion. According to their score, studies are classified as high risk (1–3 points), intermediate risk (4–5 points), or low risk of bias (6–9 points). The evidence was graded according to the recommendations of the Grading of Recommendations Assessment, Development and Evaluation working group by two authors via GRADEpro software (GRADEpro GDT, Evidence Prime Inc., Hamilton, ON, Canada), which is available at https://gdt.gradepro.org/. The presence of publication bias was investigated by visual inspection of the funnel plots and Egger’s test.

### 2.3. Statistical Analysis

Meta‐analysis was performed via Review Manager Software (RevMan®, The Cochrane Collaboration, version 5.4.1). The generic inverse variance (IV) method was used to analyze the investigated outcomes. The results from each study are reported as the means and standard deviations (SDs). Analysis was performed via the random effect (RE) model; *p* values were two‐tailed and considered significant if they were < 0.05. The presence of statistical heterogeneity was assessed via the X2 (Cochran *Q*) test. Heterogeneity was likely if *Q* > degrees of freedom (df) was suggested and confirmed if *p* ≤ 0.10. Quantification of heterogeneity was performed, and values of I^2^ ranging from 0% to 24.9%, 25%–49.9%, 50%–74.9%, and > 75% were considered none, low, moderate, and high heterogeneity, respectively. We planned subgroup analyses to explore potential sources of heterogeneity, including (a) continuous catheter techniques versus single‐shot blocks and (b) type of background anesthesia (general versus spinal). These analyses were performed using the test for subgroup differences in Review Manager with statistical significance set at *p* < 0.10. Moreover, we performed two sensitivity analyses using the leave‐one‐at‐time approach and removing the studies at high risk of bias. We conducted TSAs to evaluate the robustness of our findings, calculating the information size (the power of the meta‐analysis) for the three investigated outcomes. We used freely available TSA software (0.9.5.10 Beta Version; Copenhagen Trial Unit’s TSA Software®; Copenhagen, Denmark). The information size was computed assuming an alpha risk of 5% and a beta risk of 20%. The estimated effects were computed by averaging the results of the classical meta‐analysis method. Further details on the TSA and its interpretation are available elsewhere [21, 22].

## 3. Results

**FIGURE 1:**
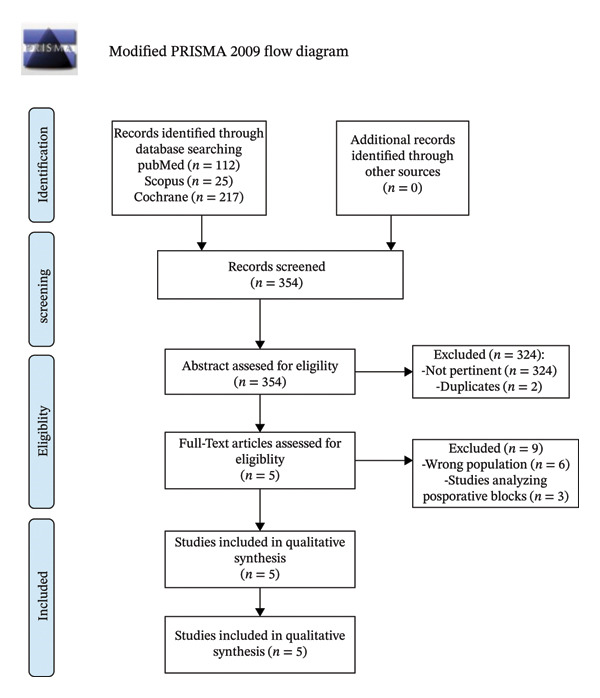
PRISMA flowchart.

Our systematic search identified 354 findings, 112 of which were from PubMed, 25 from Scopus, and 217 on Cochrane Library. No other findings were retrieved manually. As shown in the PRISMA flow diagram (Figure 1), after the evaluation of all the abstracts, 14 full‐text articles were assessed against the PICOS criteria.

Nine papers were excluded because of the study design: in six studies included patients did not undergo hip arthroplasty, and in the other three papers, patients underwent PENG block postoperatively. The remaining 5 RCTs were included in our meta‐analysis [13, 23–26], all reporting data on opioid consumption in the first 24 h. All included studies reported opioid consumption standardized to morphine milligram equivalents (MME) or used morphine directly as the primary opioid [13, 23–26]. Details of opioid measurement and conversion approaches for each study are provided in Supporting Information 3. The characteristics of the included studies are reported in Table 1.

**TABLE 1:** Characteristics of the included studies.

Study, year, design	Population	Intervention	Comparator	Primary outcome	Reference
Duan et al., 2023, Prospective RCT	Adults undergoing THA	Continuous PENG block	Continuous FICB	Pain scores and quadriceps muscle strength	[23]
Liang et al., 2023, Prospective RCT	Adults undergoing THA	PENG block	S‐FICB	Cumulative sufentanil consumption at 24 h	[24]
Choi et al., 2022, Prospective RCT	Adults undergoing THA	PENG block	S‐FICB	Postoperative opioid consumption	[25]
Vamshi et al., 2023, Prospective RCT	Adults undergoing THA	PENG block	S‐FICB	Dynamic pain scores at 24 h	[26]
Aliste et al., 2021, Prospective RCT	Adults undergoing THA	PENG block	S‐FICB	Opioid consumption within 24 h postoperatively	[16]

*Note:* PENG: pericapsular nerve group, S‐FICB: suprainguinal fascia iliaca compartment block.

Abbreviations: RCT = randomized controlled trial, THA = total hip arthroplasty, VAS = visual analog scale.

Among the included studies, four compared PENG block to S‐FICB [13, 24–26], and one compared continuous PENG block to continuous S‐FICB [23]. Given the substantial heterogeneity observed in the primary outcome and the inclusion of one study employing continuous catheter technique, we performed a subgroup analysis comparing continuous PENG block versus single‐shot PENG blocks.

### 3.1. Opioid Consumption in the First 24 h

The analysis of opioid consumption in the first 24 h included five studies, four of which were in the subgroup with a single shot of S‐FICB as a comparison, while one compared a continuous PENG block to a continuous S‐FICB. All studies reported opioid consumption in the first 24 h after surgery.

**FIGURE 2:**
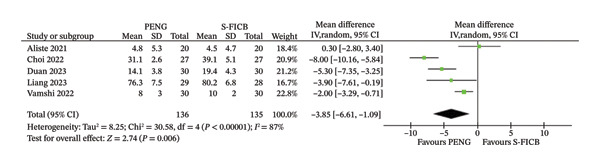
Forest plot for opioid consumption in the first 24 h after surgery.

In the overall analysis, treatment with the PENG block in patients undergoing spinal or general anesthesia for hip arthroplasty was associated with reduced opioid consumption compared with S‐FICB: MD −3.85 (95% CI −6.61, −1.09), *p* = 0.006; high heterogeneity (*I* ^2^ = 87%; Figure 2). The continuous catheter subgroup (Duan et al., *n* = 160) demonstrated a larger effect size (MD −6.32 mg, 95% CI −7.91 to −4.73) compared to the single‐shot subgroup (four studies, *n* = 664; MD −3.18 mg, 95% CI −6.38 to 0.01, *I* ^2^ = 89%). The test for subgroup differences was statistically significant (*p* = 0.009), indicating that catheter‐based continuous infusion may provide enhanced opioid‐sparing effects. However, substantial heterogeneity persisted within the single‐shot subgroup (*I* ^2^ = 89%), suggesting that factors beyond catheter technique contribute to variability in effect sizes.

### 3.2. Pain Scores (Visual Analog Scale [VAS]) at 24 h

Five studies were included in the analysis of pain scores at 24 h, reported as VAS. Among them, four had one shot S‐FICB as a comparison, while one compared continuous PENG block to continuous S‐FICB. The 24‐h timepoint was selected because it captures peak postoperative pain, represents the critical period for opioid‐sparing effects enabling mobilization, and aligns with standardized enhanced recovery protocols. All studies reported VAS scores at 24 h after surgery.

**FIGURE 3:**
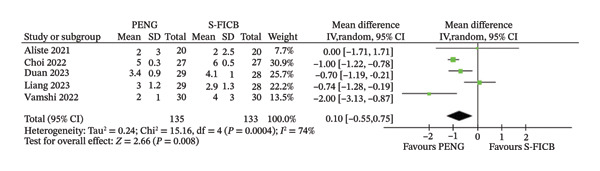
Forest plot for pain scores (visual analog scale) at 24 h.

In the overall analysis, treatment with PENG block in patients undergoing spinal or general anesthesia for hip arthroplasty was associated with reduced VAS as compared to S‐FICB: MD −0.74 (95% CI −1.28, −0.19), *p* = 0.008; moderate heterogeneity (*I* ^2^ = 74%; Figure 3). When performing the subgroup analysis, similar pattern emerged, with the continuous catheter technique showing MD −1.10 (95% CI −1.54 to −0.66) versus single‐shot MD −0.62 (95% CI −1.26 to 0.02, *I* ^2^ = 76%). The test for subgroup differences approached but did not reach statistical significance (*p* = 0.11). Again, heterogeneity remained moderate‐to‐high within the single‐shot subgroup.

### 3.3. Time to Mobilization

**FIGURE 4:**
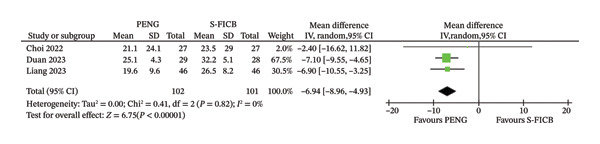
Forest plot for time to mobilization.

The analysis of opioid consumption included only three studies. In the overall analysis, treatment with PENG block was associated with a reduced time to mobilization (MD −6.94 (95% CI −8.96, −4.93), *p* < 0.00001, with no heterogeneity (*I* ^2^ = 0%), as reported in Figure 4.

### 3.4. Quality Assessment, Publication Bias, and GRADE of Evidence

The assessment of risk of bias via the Cochrane RoB 2.0 tool revealed that three studies [13, 24, 25] demonstrated a low risk of bias across all domains, reflecting robust methodological quality with appropriate randomization, blinding procedures, complete outcome data, and comprehensive reporting (Supporting Information 4). The remaining two studies [23, 26] were assessed as having some concerns, primarily due to limitations in blinding procedures for outcome assessment or incomplete reporting of allocation concealment. No studies were classified as having a high risk of bias. Visual inspection of funnel plots suggested no obvious publication bias; however, with only five studies included, this assessment has limited statistical power and should be interpreted cautiously.

The quality of evidence was assessed via GRADEpro software for all three investigated outcomes. For time to mobilization, the certainty of evidence was rated as high, with consistent findings across studies and no heterogeneity (*I* ^2^ = 0%). The evidence for reduced opioid consumption at 24 h was rated as moderate, with a downgrade primarily due to serious inconsistency across studies. For pain scores at 24 h, the certainty of evidence was rated as moderate, with a downgrade due to very serious inconsistency. No serious concerns were identified regarding risk of bias, indirectness, imprecision, or publication bias across all outcomes (Supporting Information 5).

### 3.5. Sensitivity Analyses

The sensitivity analysis according to the leave‐one‐at‐time approach for the primary outcome did not change the overall results, with values ranging from *p* = 0.02 and *p* = 0.002. The same results were observed for the time to mobilization outcome, with a *p* value always less than 0.0002. However, the same approach conducted for the pain score outcome (VAS) yielded a *p* = 0.12 and a *p* = 0.07 when the studies by Choi et al. [25] or Duan et al. [23] were removed.

### 3.6. TSAs

We performed TSAs for all three outcomes. For opioid consumption, the required information size was not fully reached (271/289 patients, 94%), and while the *Z*‐curve crossed the alpha‐spending boundary, the high heterogeneity (*I* ^2^ = 87%) limits conclusiveness (Supporting Information 6). Similar findings were observed for pain scores (268/300 patients, 89%; *I* ^2^ = 74%) (Supporting Information 7). Only time to mobilization achieved full information size (203/35 patients) with no heterogeneity (*I* ^2^ = 0%), providing the most robust evidence among outcomes analyzed.

## 4. Discussion

To the best of our knowledge, this is the first systematic review and meta‐analysis of RCTs evaluating the efficacy of PENG block compared with S‐FICB in patients undergoing hip arthroplasty. Our results showed that PENG block was superior to S‐FICB in terms of opioid administration in the first 24 h, VAS at 24 h, and time to mobilization.

The primary endpoint of our analysis, opioid consumption within the first 24 h after surgery, was significantly lower with PENG block than S‐FICB. This finding is particularly relevant since opioid consumption is associated with fewer complications, such as headaches, PONV, constipation, and hypoxemia, and an increased risk for chronic drug abuse over time. However, in our examination, these findings were affected by high sample heterogeneity (*I* ^2^ = 87%), which indicates that studies have different effect sizes, possibly because of the lack of individual preoperative analgesic management protocols, surgical methods, and methods used to assess outcomes.

Similarly, the VAS score at 24 h was significantly lower in the PENG block group than in the S‐FICB group. This finding aligns with the theoretical mechanism of PENG block, which specifically targets the articular branches of the femoral and obturator/accessory obturator nerves innervating the anterior hip capsule [10], potentially providing more focused analgesia for hip joint pain. However, the moderate heterogeneity (*I* ^2^ = 74%) and the conflicting results when performing the sensitivity analysis suggest caution when interpreting these results. Perhaps the most compelling finding of our analysis is the consistent reduction in time to mobilization associated with PENG block. With no heterogeneity (*I* ^2^ = 0%) and consistent effects across subgroups, the PENG block demonstrated a clear advantage in facilitating earlier ambulation compared with S‐FICB. This outcome is particularly relevant in the context of enhanced recovery after surgery (ERAS) protocols, where early mobilization is essential for reducing complications such as venous thromboembolism, pulmonary complications, and prolonged hospital stays [27]. The observed average reduction of more than 6 h in time to mobilization may translate to meaningful clinical benefits in terms of recovery treatments and resource utilization.

Trial sequential analysis provides differential support across outcomes. Time to mobilization demonstrated full information size with no heterogeneity, providing robust evidence. For opioid consumption and pain scores, incomplete information size (94% and 89%) combined with substantial heterogeneity (*I* ^2^ = 87% and 74%) limits conclusiveness despite alpha‐spending boundary crossings. While these findings suggest analgesic benefits are unlikely to be entirely spurious, uncertainty remains, and high heterogeneity reduces the reliability of TSA conclusions. The substantial heterogeneity observed for opioid consumption (*I* ^2^ = 87%) and pain scores at 24 h (*I* ^2^ = 74%) warrants careful consideration and limits the precision of our effect estimates. This variability likely stems from differences in local anesthetic agents and volumes (20–30 mL of ropivacaine 0.375%–0.5% or bupivacaine 0.25%), delivery technique (continuous versus single‐shot), background anesthesia (spinal versus general), perioperative analgesic protocols, and surgical approaches. As demonstrated in our subgroup analysis, continuous catheter techniques (Duan et al.) showed substantially larger effect sizes than single‐shot approaches, suggesting sustained local anesthetic delivery enhances analgesia, though heterogeneity persisted even within the single‐shot subgroup (*I* ^2^ = 89%).

An important consideration is the clinical meaningfulness of our observed effect sizes. The overall opioid reduction of −3.85 mg falls below established minimal clinically important differences (MCIDs) of 10–15 mg for orthopedic surgery; however, continuous catheter techniques achieved −6.32 mg reduction, approaching clinically meaningful thresholds. Similarly, the VAS reduction of −0.74 falls below typical MCID thresholds of 1.0–1.5 points, though continuous techniques achieved −1.10. These incremental improvements should be interpreted within the context of multimodal analgesia, where cumulative opioid‐sparing effects contribute to clinically meaningful outcomes, particularly for high‐risk patients where minimizing opioid exposure is paramount. Importantly, the 7‐h reduction in time to mobilization represents an unequivocally clinically meaningful benefit that directly impacts patient‐centered outcomes including complication risk, hospital length of stay, and functional recovery [27]. The clinical value of PENG block emerges from this synergistic benefit profile: adequate analgesia combined with reduced opioid exposure, preserved motor function, and substantially earlier mobilization, a combination that aligns with ERAS principles even when individual analgesic metrics show incremental rather than dramatic improvements. The single study employing continuous catheter techniques [23] warrants separate consideration, as catheter‐based approaches represent a fundamentally different analgesic paradigm from single‐shot techniques. Continuous PENG block demonstrated larger effect sizes for both opioid consumption (MD −6.32 vs. −3.18 mg) and pain scores (MD −1.10 vs. −0.62), approaching clinically meaningful thresholds. This suggests that sustained local anesthetic delivery may overcome the limitations of single‐shot duration and provide extended analgesia throughout the critical first 24 h postoperatively. However, with only one RCT available, these findings should be considered preliminary. Continuous catheter techniques introduce additional considerations including catheter placement complexity, infection risk, resource utilization, and patient mobility constraints that must be weighed against potential analgesic benefits. Further dedicated investigation comparing continuous PENG to continuous S‐FICB, as well as head‐to‐head comparisons between continuous and single‐shot PENG approaches, is needed before definitive recommendations can be made regarding optimal delivery techniques. Our results align with and extend previous investigations of regional analgesic techniques for hip surgery. The present systematic review updates and extends the meta‐analysis by Prakash et al. [18], which included 12 RCTs (644 patients) comparing PENG block with FICB (both suprainguinal and infrainguinal approaches combined). While their findings showed improved early analgesia and reduced 24‐h opioid consumption but no difference in 24‐h pain scores, our analysis provides three key methodological refinements. First, we exclusively compared PENG block to S‐FICB, which demonstrates superior lumbar plexus spread compared to infrainguinal approaches [16, 17] and represents the preferred technique for hip surgery [18, 19], providing more clinically specific guidance. Second, we incorporated TSAs for all outcomes [21, 22], demonstrating that sufficient evidence has been accumulated (Z‐curve crossing alpha‐spending boundaries), providing reassurance against false‐positive findings and suggesting further trials may be unnecessary for these endpoints. Third, our analysis includes recent RCTs published after April 2023, including continuous catheter techniques [23], providing preliminary evidence for extended analgesic coverage. Moreover, a Cochrane review by Guay et al. [17] concluded that peripheral nerve blocks provide improved analgesia compared with systemic analgesia for hip arthroplasty, although they did not specifically evaluate PENG block. More recent studies have suggested that PENG block may provide superior analgesia with less motor blockade than traditional approaches [11, 28]. Our findings provide systematic evidence supporting these observations and specify the comparative efficacy of PENG block against S‐FICB.

The physiological basis for the superior performance of PENG block, particularly with respect to earlier mobilization, is likely related to its targeted approach. Unlike techniques such as S‐FICB, which can cause quadriceps weakness due to blockade of the femoral nerve [13], PENG block primarily targets the articular branches innervating the hip joint while theoretically sparing motor function [10, 11]. This selective approach may explain the consistent advantage observed in time to mobilization, as patients maintain better motor function while still achieving effective analgesia.

Several limitations should be considered when our findings are interpreted. First, our stricter inclusion criteria (restricting to S‐FICB only) resulted in fewer included studies (five RCTs) compared to broader prior syntheses [15], potentially limiting generalizability but enhancing clinical specificity. Second, the relatively small number of included studies (five RCTs) limits generalizability. TSA demonstrated conclusive evidence only for time to mobilization (full information size, *I* ^2^ = 0%); incomplete information size and high heterogeneity for analgesic outcomes require cautious interpretation. Third, the high heterogeneity observed for opioid consumption within 24 h and pain scores at 24 h indicates variability in effect sizes across studies, potentially owing to differences in surgical techniques, perioperative protocols, or outcome measurement methods. Fourth, while we assessed publication bias through funnel plots, the small number of included studies limits the reliability of this assessment. Also, the included studies primarily evaluated short‐term outcomes (within 24 h), and the long‐term impact of PENG block on functional recovery and chronic pain remains to be fully explored. Additionally, while our findings demonstrate statistical significance, the modest magnitude of opioid and pain score reductions at the population level may not consistently achieve established clinical significance thresholds, though optimized protocols (continuous catheters and adequate volumes) and the substantial mobilization benefits suggest clinically relevant applications within enhanced recovery pathways.

Additional methodological limitations warrant acknowledgment. First, our restriction to English‐language publications may introduce language bias, potentially excluding relevant studies published in other languages. Second, although our search included the Cochrane Central Register of Controlled Trials (which incorporates trial registry data), we did not conduct explicit independent searches of clinical trial registries such as ClinicalTrials.gov or the WHO ICTRP.

Despite these limitations, our findings have important clinical implications. The significant reduction in opioid consumption, improved pain control, and earlier mobilization associated with PENG block suggest that this technique may be valuable in multimodal analgesic protocols for patients undergoing hip arthroplasty. Particularly in the context of ERAS pathways, where early mobilization and reduced opioid use are key goals, PENG block appears to offer advantages over conventional approaches such as S‐FICB.

## 5. Conclusions

This systematic review and meta‐analysis demonstrated that PENG block is associated with reduced opioid consumption, improved pain control, and significantly earlier mobilization than S‐FICB in patients undergoing hip arthroplasty. These findings support the integration of PENG block into multimodal analgesic strategies for hip arthroplasty, with potential benefits for enhanced recovery pathways. However, the heterogeneity observed in our analysis highlights the need for further research to optimize implementation and confirm long‐term benefits.

## Author Contributions

Luigi La Via: conceptualization, methodology, investigation, formal analysis, data curation, writing–original draft, and project administration. Fabio Raciti: investigation, data curation, validation, and visualization. Giovanni Guarneri: methodology, investigation, validation, and writing–review and editing.

Ornella Rosa: data curation, formal analysis, and visualization. Francesco Vasile: writing–review and editing, validation, and data curation. Francesco Perna: resources, validation, and writing–review and editing. Marco Sapienza: investigation, resources, and visualization. Carmelo Calvagna: software, validation, and writing–review and editing. Gianluca Testa: conceptualization, supervision, validation, and writing–review and editing. Gianluca Testa: conceptualization, supervision, resources, writing–review and editing, and project administration.

## Funding

This research did not receive any specific grant from funding agencies in the public, commercial, or not‐for‐profit sectors. Open access publishing facilitated by Universita degli Studi di Catania, as part of the Wiley ‐ CRUI‐CARE agreement.

## Disclosure

All the authors have read and agreed to the published version of the manuscript.

## Ethics Statement

The authors have nothing to report.

## Consent

The authors have nothing to report.

## Conflicts of Interest

The authors declare no conflicts of interest.

## Supporting Information

Additional supporting information can be found online in the Supporting Information section.

## Supporting information


**Supporting Information** Supporting Information 1. PRISMA Checklist. Supporting Information 2. PICOS Criteria. Supporting Information 3. Opioid Measurement and Standardization Approaches. Supporting Information 4. Summary of Risk of Bias Assessment. Supporting Information 5. Summary Table of GRADE of Evidence. Supporting Information 6. Trial sequential analysis for opioid consumption in the first 24 h. Supporting Information 7. Trial sequential analysis on VAS.

## Data Availability

The datasets used and/or analyzed during the current study are available from the corresponding author upon reasonable request.
